# Independent and joint effects of tea and milk consumption on oral cancer among non-smokers and non-drinkers: a case-control study in China

**DOI:** 10.18632/oncotarget.15096

**Published:** 2017-02-04

**Authors:** Fa Chen, Lingjun Yan, Lisong Lin, Fengqiong Liu, Yu Qiu, Fangping Liu, Jiangfeng Huang, Junfeng Wu, Lin Cai, Guoxi Cai, Kiyoshi Aoyagi, Baochang He

**Affiliations:** ^1^ Department of Epidemiology and Health Statistics, School of Public Health, Fujian Medical University, Fujian, China; ^2^ Key Laboratory of Ministry of Education for Gastrointestinal Cancer, Fujian Medical University, Fujian, China; ^3^ Department of Oral and Maxillofacial Surgery, the First Affiliated Hospital of Fujian Medical University, Fujian, China; ^4^ Nagasaki Prefectural Institute of Environmental Research and Public Health, Institute of Tropical Medicine, Nagasaki University, Nagasaki, Japan; ^5^ Department of Public Health, Nagasaki University Graduate School of Biomedical Sciences, Nagasaki, Japan

**Keywords:** oral cancer, tea, milk, non-smoker, non-drinker

## Abstract

This study aims to evaluate the independent and joint effects of tea and milk consumption on oral cancer risk among non-smokers and non-drinkers (NS/ND). A hospital-based case-control study was performed in Fujian, China. 421 cases and frequency-matched 1398 controls were included without tobacco smoking and alcohol drinking habits. Unconditional logistic regression model was used to assess the relationship of tea and milk consumption with oral cancer risk. Tea and milk consumption were significantly associated with decreased risk of oral cancer, the adjusted odds ratios (aORs) were 0.73 (95% CI: 0.54-0.97) and 0.69 (95% CI: 0.55-0.88), respectively. According to subgroup analysis, the inverse associations between tea consumption and oral cancer risk were only observed among the elders (>60 years) and urban residents. While the protect effect of milk drinking was more obvious in males, normal body mass index population (18.5–23.9), urban residents and those age ≤ 60 years. Additionally, a significantly multiplicative interaction between tea and milk consumption was observed for oral cancer risk (*P* = 0.001). The present study is the first to simultaneously assess the association of tea consumption and milk drinking with oral cancer risk. The results suggest that tea and milk consumption are independent protective factors for oral cancer among NS/ND, with a joint effect between them.

## INTRODUCTION

Oral cancer, a major subgroup of head and neck carcinoma, ranks the eighth most frequent cancer worldwide [1]. As reported, the age-standardized incidence rate of oral cancer in China was 2.22 per 100,000 and 0.9 per 100,000 as mortality rate in 2011 [2]. Tobacco smoking and alcohol drinking are considered as the main risk factors [3, 4]. However, there are not all smokers and drinkers developing oral cancer, which suggests that other factors may play potential roles in the etiology of this disease.

Tea is the most consumed beverage in China, especially for the population in Fujian province, and the major types are green tea and black tea. It is well known that tea is characterized by a high content of polyphenols which has been identified to inhabit the tumor growth and migration in animal models or human malignant cells in *vitro* [5], including the oral cancer [6]. In recent decades, with the development of dietary guidelines, milk consumption has also greatly increased in China. The anti-cancer properties of milk are mostly attributed to its calcium [7] and lactoferrin [8]. Moreover, evidences from laboratory studies also showed that milk could increase the anti-cancer effect of tea due to the interaction between epigallocatechin-3-gallate (EGCG) and lactoferrin [9].

Many epidemiologic studies have separately examined the relationship between tea or milk consumption and risk of oral cancer. Chen *et al*. [10] indicated that tea drinking reduced the risk of oral cancer, but Hildebrand *et al*. [11] observed no association. Additionally, there are also controversial conclusions about milk intake and risk of oral cancer [12, 13]. However, to date, few epidemiologic studies reported the interaction between tea and milk drinking on oral cancer. Moreover, the majority of previous studies only adjusted smoking and alcohol drinking rather than limiting the subjects to non-smokers and non-drinkers (NS/ND) [14, 15], which may not completely eliminate the potential confounding effects of them.

Therefore, we conducted a hospital-based case-control study among NS/ND in China, aiming to elucidate the role of tea and milk consumption in oral cancer and further explore the joint effect of tea drinking and milk consumption on oral cancer.

## RESULTS

The distribution of 421 cases and 1398 controls by main characteristics is presented in Table 1. Cases and controls were similar in the distributions of age, gender and residence (*P* > 0.05), but significantly differed in education level, marital status, BMI, denture wearing, tea and milk consumption (*P* < 0.05). The majority of subjects in this study were females, who accounted for 75.06% cases and 71.26% controls, respectively. Compared with oral cancer patients, controls were more likely to have tea consumption, although the proportions of tea drinking were low in both groups (23.68% vs.18.05%). And milk drinking also showed a higher percentage among control subjects.

**Table 1:** Main characteristics of case and control subjects

Variables	Cases (%)	Controls (%)	*P*-value
	(*n* = 421)	(*n* = 1398)	
Age(years)			0.114
≤60	218(51.78)	785(56.15)	
>60	203(48.22)	613(43.85)	
Gender			0.126
Male	105(24.94)	402(28.76)	
Female	316(75.06)	996(71.24)	
Residence			0.112
Rural	242(57.48)	742(53.08)	
Urban	179(42.52)	656(46.92)	
Education level			<0.001
Primary and below	191(45.37)	593(42.42)	
Middle school	174(41.33)	448(32.05)	
College and above	56(13.30)	357(25.54)	
Marital status			<0.001
Married	374(88.84)	1132(80.97)	
Other	47(11.16)	266(19.03)	
BMI (kg/m2)			<0.001
18.5-23.9	259(61.52)	809(57.87)	
<18.5	52(12.35)	102(7.30)	
≥24	110(26.13)	487(34.84)	
Denture wearing			<0.001
No	216(51.31)	873(62.45)	
Yes	205(48.69)	525(37.55)	
Tea consumption			0.015
No	345(81.95)	1067(76.32)	
Yes	76(18.05)	331(23.68)	
Milk consumption			<0.001
No	254(60.33)	685(49.00)	
Yes	167(39.67)	713(51.00)	

Table 2 shows the relationship between tea and milk consumption and risk of oral cancer among NS/ND. After adjustment for potential confounders, tea and milk consumption were significantly associated with decreased risk of oral cancer, the adjusted odds ratios (aORs) were 0.73 (95% CI: 0.54-0.97) and 0.69 (95% CI: 0.55-0.88), respectively. When stratified by age, gender, residence and BMI, a stronger protective effect of tea drinking was observed in older age group (>60 years) or urban residents. For milk intake, there were statistically significant reduced risk of oral cancer in men, normal mass index population (BMI) population, urban residents and those age ≤ 60 years.

**Table 2:** Associations between tea and milk consumption and risk of oral cancer

Variables	Tea consumption				Milk consumption			
	No		Yes		No		Yes	
	Cases/ Controls	*OR*	Cases/ Controls	*OR*(95%*CI*)^a^	Cases/ Controls	*OR*	Cases/ Controls	*OR*(95%*CI*)^a^
All subjects	345/1067	1.00	76/331	0.73(0.54-0.97)	254/685	1.00	167/713	0.69(0.55-0.88)
Age(years)								
≤60	172/602	1.00	46/183	0.86(0.58-1.28)	137/334	1.00	81/451	0.61(0.44-0.85)
>60	173/465	1.00	30/148	0.46(0.29-0.73)	117/351	1.00	86/262	0.82(0.59-1.16)
Gender								
Male	73/252	1.00	32/150	0.68(0.42-1.11)	67/184	1.00	38/218	0.56(0.35-0.89)
Female	272/815	1.00	44/181	0.74(0.51-1.06)	187/501	1.00	129/495	0.75(0.57-0.98)
Residence								
Rural	200/626	1.00	42/116	1.03(0.69-1.55)	160/436	1.00	82/306	0.78(0.57-1.07)
Urban	145/441	1.00	34/215	0.46(0.30-0.71)	94/249	1.00	85/407	0.55(0.39-0.78)
BMI								
18.5-23.9	215/631	1.00	44/178	0.71(0.48-1.04)	156/370	1.00	103/439	0.62(0.46-0.84)
<18.5	43/87	1.00	9/15	0.95(0.35-2.57)	28/35	1.00	24/67	0.54(0.25-1.17)
≥24	87/349	1.00	23/138	0.70(0.41-1.18)	70/280	1.00	40/207	0.80(0.52-1.24)

^a^ORs were adjusted for age, gender, education level, marital status, residence, body mass index and denture wearing.

**Figure 1:**
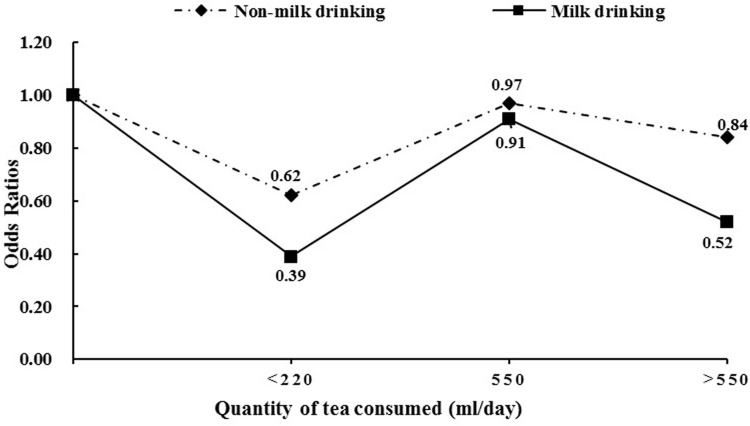
Quantity of tea consumed (ml/day) and the risk of oral cancer stratified by milk drinking status

When stratified by milk consumption, the associations between tea consumption and oral cancer risk were different (Table 3). In milk drinking population, 207 participants (23.5%) were reported a habit of tea consumption, and tea drinkers had the lower risk of oral cancer than non-tea drinkers (aOR=0.62, 95% CI: 0.39-0.98). Moreover, duration of tea consumption <20 years, quantity of tea consumed (ml/day) <500, weak concentration of tea consumed and green tea intake were at decreased risk for oral cancer. However, no associations were observed between tea-related variables and oral cancer risk among non-milk drinkers. Furthermore, the quantity of tea consumed (ml/day) was categorized into four groups based on tertiles of controls (zero, <220 ml/day, 220-550 ml/day, and >550 ml/day). And then, overall, the ORs of tea consumption from zero to more than 550 ml/day were lower among milk drinkers than non-milk drinkers. But there were not significant trends for the risk with the increasing amount of tea consumed in two groups (*P*_trend_ >0.05, Figure 1).

**Table 3:** Tea consumption and the risk of oral cancer stratified by milk drinking status

Variables	Milk drinking		Non-milk drinking	
	Cases/Controls	*OR*(95%*CI*)^a^	Cases/Controls	*OR*(95%*CI*)^a^
Tea consumption				
No	139/534	1.00	206/533	1.00
Yes	28/179	0.62(0.39-0.98)	48/152	0.81(0.55-1.19)
Duration of tea consumption (years)				
No	139/534	1.00	206/533	1.00
<20	10/100	0.42(0.21-0.84)	21/69	0.72(0.42-1.24)
≥20	18/79	0.84(0.48-1.48)	27/83	0.89(0.55-1.44)
Quantity of tea consumed (ml/day)				
No	139/534	1.00	206/533	1.00
<500	13/104	0.49(0.27-0.92)	26/77	0.85(0.52-1.38)
≥500	15/75	0.79(0.43-1.46)	22/75	0.77(0.46-1.31)
Concentration of tea consumed				
No	139/534	1.00	206/533	1.00
Weak	10/75	0.48(0.24-0.98)	17/54	0.78(0.44-1.40)
Moderate	15/88	0.68(0.38-1.23)	21/75	0.77(0.46-1.29)
Strong	3/16	0.73(0.21-2.56)	10/23	1.15(0.54-2.48)
Types of tea				
No	139/535	1.00	206/534	1.00
Green tea	14/100	0.55(0.30-0.99)	26/76	0.91(0.56-1.48)
Oolong tea	9/41	0.90(0.42-1.96)	13/43	0.76(0.40-1.47)
Others	5/37	0.47(0.18-1.25)	9/32	0.77(0.36-1.66)

^a^ORs were adjusted for age(y), gender, education level, marital status, residence, body mass index and denture wearing.

The joint effect of tea and milk consumption is shown in Table 4. On the comparison of those without tea and milk intake, participants who regularly consumed tea and milk showed the lowest risk of oral cancer (aOR=0.43, 95% CI: 0.28-0.67). Moreover, there was a significantly multiplicative interaction between tea and milk consumption on the risk of oral cancer. (OR_multiplicative_=0.50, 95% CI=0.33-0.76; *P* = 0.001). However, the significantly additive interaction was not observed (RPRI: -0.19, 95% CI: -0.60 to 0.23; AP: -0.44, 95% CI: -1.45 to 0.58; SI: 1.49, 95% CI: 0.51 to 4.35; data not shown).

**Table 4:** Joint effect of tea and milk drinking on oral cancer risk

Variables		Cases(%) n= 421	Controls(%) n= 1398	*OR*(95%*CI*)^a^
Tea drinking	Milk drinking			
No	No	206(48.93)	533(38.13)	1.00
No	Yes	139(33.02)	534(38.20)	0.75(0.58-0.97)
Yes	No	48(11.40)	152(10.87)	0.87(0.60-1.26)
Yes	Yes	28(6.65)	179(12.80)	0.43(0.28-0.67)

^a^ORs were adjusted for age(y), gender, education level, marital status, residence, body mass index and denture wearing.

## DISCUSSION

Overall, in this hospital-based case-control study, tea or milk consumption showed a protective effect against oral cancer among NS/ND. Furthermore, the beneficial effect of tea consumption was only observed in milk drinkers but not in non-milk drinkers. Additionally, a significantly multiplicative interaction was found between tea and milk consumption on oral cancer risk.

Several previous epidemiological investigations have also demonstrated that tea consumption could reduce the risk of oral cancer [15, 16]. Evidence from laboratorial studies have identified that the anti-oxidant and anti-carcinogenic components of tea are mostly attributed to polyphenols (especially EGCG), with the inhibitory effects on proliferation and invasion of tumor cells [17, 18]. Interestingly, when stratified by select demographic characteristics, the negative correlations between tea drinking and oral cancer risk were more obvious in elders (>60 years) and urban residents, which is similar to a recent study [10]. However, the potential mechanisms suggested to explain these findings remain to be clarified.

Milk is well-known good source of calcium, dietary vitamin D and proteins that provides biological activities from anti-microbial effect [19], immunomodulatory activity [20] to anti-carcinogenic function [21], and thus contributes to human health. Nevertheless, results from epidemiological studies on the association between milk drinking and oral cancer risk are not consistent. A case-control study conducted in Swiss showed an inverse relation between milk intake and oral cancer risk [12], which is consistent with our result. Whereas a significantly increased risk was found by Bravi *et al*. [13]. The possible reasons for the inconsistencies may be owing to differences in study population and adjustment of covariates.

Adding milk to tea is a common practice in China. In this study, a significantly multiplicative interaction was observed between tea and milk consumption on the risk of oral cancer. A cellular experiment indicated that addition of lactoferrin to tea polyphenols had stronger effect on inhibition of tongue squamous cell proliferation than tea polyphenols alone [9]. Mohan *et al*. [6] also obtained the similar results in buccal pouch carcinogenesis by animal model. This finding provides a new information that tea drinking with the addition of milk may reach to the greater benefit on the prevention of oral cancer.

In the present work, valid selection of all subjects was based on non-smoking and non-drinking population, helping to eliminate the confounding interferences of tobacco and alcohol. However, it also had several limitations. Firstly, recall bias is unavoidable in retrospective studies, but tea and milk consumption are not recognized to be associated with oral cancer in general, thus this possible bias would be non-differential among cases and controls. Secondly, since all subjects were recruited from only one hospital, selection bias should be concerned. However, the participation rates of cases and controls were high and all eligible subjects were based on strict criteria, which may reduce the possibility of selection bias. Thirdly, the amount constraints of these beverages consumed among NS/ND may weaken the authenticity and reliability of this study to a certain. Therefore, a further study with larger sample size is required to confirm the results of this work.

In conclusion, this hospital-based case-control study is the first to simultaneously assess the relationship of tea consumption and milk drinking with oral cancer risk. Our results suggest that tea and milk consumption are independent protective factors for oral cancer among NS/ND with a synergistic effect between them. The findings may provide a new strategy for oral cancer prevention.

## MATERIALS AND METHODS

### Study participants

From September 2010 to September 2016, we performed a hospital-based case-control study on oral cancer in Fujian province, China. As described previously [22], participants were recruited from the First Affiliated Hospital of Fujian Medical University and all subjects were NS/ND. Cases were defined as histologically confirmed primary oral carcinomas with no previous history of chemotherapy and radiotherapy. A total of 421 cases (median age 58 years, range: 20-91) were included in the final analysis. Meanwhile, 1398 controls (median age 54 years, range: 20-89) were recruited among inpatients and outpatients without a diagnosis of cancer, and frequency-matched by age (±3 years) and gender with cases. The subjects in control group included (1) healthy population; (2) ear and eye disorders; (3) skin and subcutaneous tissue disorders; (4) trauma; (5) upper-respiratory tract diseases; (6) gastro-intestinal disorders. The proportion of rejection in this study was less than 5%. This study was approved by the Institutional Review Board of Fujian Medical University (Fuzhou, China).

### Data collection

Face to face interviews were conducted by well-trained interviewers with the structured questionnaire to collect information, including socio-demographic characteristics, smoking and alcohol drinking history, tea and milk consumption habits, diet habits, oral health status and family history of cancer.

A person who had smoked less than 100 cigarettes and had not drunk as much as once a week in a lifetime was defined as never-smoker and never drinker. Tea consumption refers to drank at least 1 cup per week continuously for at least 6 months. Furthermore, the data on duration of tea-drinking habit (years), quantity of tea consumed (ml/day), concentration of tea (weak/moderate/strong) and types of tea (green tea/oolong tea/others), were obtained. The concentration of tea was evaluated based on the volume occupied by the brewed tea leaves in the cup (weak, the volume less than 25% of the cup; moderate, 25%-50%; and strong, >50%).

### Statistical analysis

Chi-square test was used to compare the distribution of demographic characteristics as well as tea and milk consumption between case and control groups. The associations of tea and milk drinking with oral cancer were estimated by odds ratios (ORs) and the corresponding 95% confidence intervals (CIs) derived from unconditional logistic regression model, and subgroup analysis was conducted by age group, gender, residence and BMI. Furthermore, we assessed the relationships between tea-related variables and oral cancer stratified by milk consumption. Multiplicative interaction between tea and milk consumption on oral cancer risk was also evaluated by unconditional logistic regression model. Additive interaction was assessed using the relative excess risk due to interaction (RERI), attributable proportion (AP), and synergy index (SI). The null values of RERI and AP were 0, while that of SI was 1. *P* values <0.05 (two-tailed) were considered significant. All analyses were conducted using R software version 3.1.1.
